# Pace of life predicts parasite resistance and fecundity tolerance, but not mortality tolerance, among Trinidadian guppies, *Poecilia reticulata*

**DOI:** 10.1093/evlett/qrag011

**Published:** 2026-03-31

**Authors:** Sadie Evanov, Faith Rovenolt, Natalia Tepox-Vivar, Rina Zambetti, Kelley Troutman, Vineet Nayak, Kaylee Mulligan, Saisreetha Midde, Noah T Leith, Jessica F Stephenson

**Affiliations:** Department of Biological Sciences, University of Pittsburgh, Pittsburgh, United States; Department of Biological Sciences, University of Pittsburgh, Pittsburgh, United States; Doctorado en Ciencias, Instituto de Ecología, A.C., Red de Biología Evolutiva, Xalapa, Veracruz, Mexico; Department of Biological Sciences, University of Pittsburgh, Pittsburgh, United States; Department of Biological Sciences, University of Pittsburgh, Pittsburgh, United States; Department of Biological Sciences, University of Pittsburgh, Pittsburgh, United States; Department of Biological Sciences, University of Pittsburgh, Pittsburgh, United States; Department of Biological Sciences, University of Pittsburgh, Pittsburgh, United States; Department of Biological Sciences, University of Pittsburgh, Pittsburgh, United States; Department of Environmental Science, Policy, and Management, University of California Berkeley, Berkeley, United States; Department of Biological Sciences, University of Pittsburgh, Pittsburgh, United States; Department of Zoology, Stockholm University, Stockholm, Sweden

**Keywords:** parasite resistance, mortality tolerance, fecundity tolerance, pace of life, life history, epidemic feedback

## Abstract

Host defense against parasites can include limiting parasite growth, “resistance,” limiting the mortality cost of infection, “mortality tolerance,” and limiting the reproductive cost of infection, “fecundity tolerance.” Theoretically, these 3 host strategies have very different epidemiological and evolutionary outcomes. In particular, because mortality tolerance increases parasite population size, it is under strong positive frequency-dependent selection and may therefore be less variable between populations than either resistance or fecundity tolerance. Additionally, host investment in each strategy can be expected to differ between populations that experience different ecological conditions. Here, we tested how populations of Trinidadian guppies *Poecilia reticulata* from the upper and lower courses of 3 rivers responded to experimental infection with a novel strain of *Gyrodactylus turnbulli*. In line with theoretical predictions, we found that lower course populations, previously shown to have faster paces of life, invested less in resistance and fecundity tolerance—but not mortality tolerance—than the upper course populations with slower paces of life. Our results indicate that this host–parasite interaction both conforms to evolutionary-epidemiological theoretical predictions, and is shaped by broader ecological conditions.

## Teaser

How hosts defend themselves against parasites can increase or decrease the size of the parasite population, and thus future infection risk. This epidemiological feedback may therefore shape host investment across elements of defense, which include limiting parasite growth (“resistance”), and limiting the reproductive and mortality costs of infection (“fecundity” and “mortality tolerance”, respectively). In parallel, investment in these defenses should be dictated by host life history. These ideas are well-established theoretically, and our results suggest that they shape guppy, *Poecilia reticulata*, investment in defense against *Gyrodactylus turnbulli*. Guppy populations with faster paces of life invested less in resistance and fecundity tolerance than those with slower paces of life, in line with life-history theory. However, the populations did not differ in mortality tolerance, conforming to theoretical predictions: variation in mortality tolerance should be low, as longer-lived hosts transmit parasites for longer, creating positive frequency-dependent selection for this defense strategy.

## Introduction

In this epoch of infectious disease emergence and ecological change, understanding the evolution of host defenses, particularly across ecological contexts, is essential (Seal et al., 2021). Host defense against parasites can take several forms, each with its own implications for host and parasite fitness, and thus evolutionary and coevolutionary dynamics (Kutzer et al., 2023). Such defenses are typically categorized as either “resistance,” if hosts limit parasite growth, or “tolerance,” if hosts limit the fitness cost of infection without affecting parasite growth. Intuitively, these two broad categories of defense should have different epidemiological and evolutionary implications (Miller et al., 2005; Råberg et al., 2009; Restif & Koella, 2004). Specifically, as resistance reduces parasite fitness and thus disease prevalence, selection on resistance is negatively frequency dependent. The implications of tolerance depend on the type: “fecundity tolerance,” in which hosts limit the reproductive costs of infection, does not necessarily impact disease prevalence. By contrast, “mortality tolerance,” in which hosts limit the survival costs of infection, may increase disease prevalence as infected hosts live—and can therefore transmit for—longer, leading to positive frequency dependence. Epidemiological feedbacks may therefore promote diversity in resistance, not affect diversity in fecundity tolerance, and limit diversity in mortality tolerance. These predictions have been broadly supported by theoretical (Best et al., 2008, 2014; Boots & Bowers, 1999) and empirical work across systems (Kutzer et al., 2023; Parker et al., 2014; Roy & Kirchner, 2000).

In parallel, the broader community with which both host and parasite may interact can importantly affect the evolutionary trajectory of host defenses. In particular, predation pressure may constrain or redistribute host investment in defense against parasites through at least two pathways. First, high predation pressure can favor individuals that maximize resource allocation into fecundity and reproduction early in life (i.e., “fast” pace of life) (Gadgil & Bossert, 1970; Reznick et al., 1990), at the expense of reduced investment in both resistance (Pap et al., 2014; Sparkman & Palacios, 2009) and tolerance (Johnson et al., 2012; Stephenson et al., 2015a) of parasites, in line with predictions (Ricklefs & Wikelski, 2002; van Boven & Weissing, 2004). Populations with fast life histories may thus show weaker fecundity tolerance even though they have evolved increased fecundity overall. Second, high-predation environments select for antipredator behaviors like social grouping, which may increase the opportunity for parasite transmission, leading to increased parasite population size (Walsman et al., 2022) and therefore stronger selection for investment in host defenses. Thus, the outcome of selection on host defenses due to epidemiological feedbacks likely depends on the ecological context hosts experience—of which predation pressure is a key component.

Here, we used females of the guppy, *Poecilia reticulata*, from Trinidadian populations under different predation regimes, and its gyrodactylid parasite *Gyrodactylus turnbulli*, to explore patterns of diversity in parasite resistance, fecundity tolerance, and mortality tolerance. Due to waterfalls that block the upstream migration of aquatic predators, guppies from the upper courses of Trinidadian rivers typically experience lower predation pressure than the lower courses (Magurran, 2005; Reznick et al., 1990). This difference has resulted in evolved divergent life-history strategies between upper and lower course guppy populations: lower course guppies tend to have faster paces of life than upper course guppies (Magurran, 2005; Reznick et al., 1990). Field surveys show that lower course guppies are more likely to be infected, with more individual *Gyrodactylus* spp. (Stephenson et al., 2015b), and that infected fish are in worse condition than uninfected fish in lower but not upper course populations (Stephenson et al., 2015a), consistent with slower pace of life populations investing more in both resistance and tolerance. However, these studies leave unclear whether their findings reflect population-level differences in host defense, or the fact that lower course *Gyrodactylus* spp. are more virulent (Walsman et al., 2022). Therefore, to understand population-level variation in host defenses, and how this might follow theoretical predictions across ecological contexts, we present a common-garden, holistic comparison of host defenses across Trinidadian guppy populations.

## Methods

This work was approved by the University of Pittsburgh’s Institutional Animal Care and Use Committee (IACUC), protocol 24065067.

### Fish and parasite origin and maintenance

For this study we used female guppies only: as guppies are internally fertilizing livebearers, fecundity tolerance is difficult to measure among males in this system. Females were laboratory-reared descendants of guppies collected from the upper (*n* = 19; 3 rivers) and lower (*n* = 27; 2 rivers) courses of the Guanapo, Aripo, and Yarra rivers in Trinidad in March 2020. Lower Yarra was not available. Stocks were housed and bred in mixed sex 75-L tanks on a recirculating system (Aquaneering) with daily 20% water exchanges, 12-hr L:D light cycles. The fish were fed daily with Tetramin flake and *Artemia*. Water hardness, pH, conductivity, and temperature were monitored daily, and kept at 117–135 ppm, 7.2–7.8 pH, 550–750 μS, and 25 ± 1 °C, respectively. Prior to the experiment, females were removed from stock tanks and housed individually in 1.8-L tanks under the same conditions. These individual tanks were transparent, permitting visual contact between fish to partially mitigate some of the stress associated with single housing this social species. As *G. turnbulli* is directly transmitted, we could not house fish in groups because we would not then be able to discriminate between parasite growth on individual hosts (essential for quantifying resistance and tolerance), and transmission among them.

To establish our parasite line, we transferred a single *G. turnbulli* individual from a commercially obtained guppy to a mixed stock, naïve, laboratory-bred host. We maintained this line on a group of naive hosts—“culture fish”—in a 1.8-L tank under standard conditions as above. Culture fish were screened twice a week under a dissection microscope, and naïve fish were added as needed to maintain the parasite population. We monitored culture fish closely for symptoms of gyrodactylosis; in accordance with the humane endpoints in our IACUC protocol, we euthanized fish that exhibited fused fin rays, difficulty swimming, and loss of appetite using an overdose of the anesthetic tricaine methanesulfonate (“MS222”; 4 g/L, buffered to pH 7.2 with NaHCO_3_).

### Experimental infections

Experimental infections were conducted between September 12 and November 13, 2023, in three batches. On day 0 of each batch, each experimental fish was anesthetized using MS222 in a crystallizing dish. Once immobile, fish were oriented laterally with their left side facing upward and a photograph including a ruler was taken using a mobile phone camera. These images were subsequently used to measure the fish standard length to the nearest 0.1 mm in ImageJ (Schneider et al., 2012). The fish was then poured onto a paper towel, gently dried on both sides, and weighed to the nearest 0.001 g. Next, the fish was placed back in a crystallizing dish in close contact with a recently euthanized, highly infected donor culture fish until approximately two worms transmitted (mean ± *SEM*: 1.97 ± 0.14), observed under a dissecting microscope. After infection, fish recovered from anesthesia in 1.8 L tanks in which they were kept individually under standard conditions for the remainder of the experiment. Wastewater passed through multiple filtration systems before being distributed to other tanks, preventing parasite transmission between them.

We quantified parasite loads every Monday, Wednesday, and Friday from day 1 to day 17. Fish were anesthetized for no longer than a total of 5 min for each count. Under a dissecting microscope, we manipulated each fish using a micropipette tip to accurately count the parasites on all body parts. As we were collecting data on resistance and mortality tolerance, and in accordance with our IACUC protocol, we permitted infections to proceed on experimental fish showing more severe symptoms of gyrodactylosis than our culture fish. After counts, fish were moved to fresh water and returned to their individual tanks once they had recovered from anesthesia. Fish that died before the end of the experiment were individually preserved in 70% ethanol. After counts on day 17, fish were weighed and measured as described above, and euthanized using an overdose of MS222 before being preserved in 70% ethanol.

### Dissections

To quantify the number of offspring the experimental females were carrying, we dissected them in crystallizing dishes under a microscope. Using dissection scissors, we made a small incision behind the gravid spot on the ventral side of the female and extended to the bottom of the operculum. Two shallow vertical cuts were made on either end of the incision on the dorsal side of the female to create an opening into the abdominal cavity of the fish and expose the uterus, as is standard in the literature (Martyn et al., 2006). Any embryos were removed from the uterus and counted.

### Data analysis

All analyses were conducted using R statistical software (v 4.4.0) (R Core Team, 2024). We used *gpairs* (Emerson & Green, 2025) to visualize the data, *DHARMa* (Hartig, 2022) to assess model fit, *car* (Fox & Weisberg, 2019) and *emmeans* (Lenth, 2024) to extract statistics, and *visreg* (Breheny & Burchett, 2017) and *ggplot2* (Wickham, 2016) to plot the figures. For each of the three models described below, we began with a full model including all of the fixed effects listed below, and then used the “drop1” function to reach a final model. The supplementary material includes more details and the complete code and output.

We used the data to address three questions. For the first, “do the courses differ in resistance?,” we used a linear mixed-effects model (LMM) with the area under the curve of infection load over time up until day 11 of infection as the response variable (“infection severity,” using the auc function, type = linear, from the *MESS* package [Ekstrøm, 2023]; Section 4 of the supplementary material). We log-transformed this variable as it improved the model fit. Advantages of using this metric of infection severity include that it summarizes both the duration and intensity of an individual’s infection, and that—because it uses multiple independent counts of parasite load per host—it is more robust to counting errors than would be a single count. We used infection severity up until day 11 of infection because it provides sufficient individual-level data to make this metric robust and informative, while limiting the number of fish with missing data due to premature death (see Section 4.1 of the supplementary material for more detail).

As fixed effects in the full “resistance” model, we included body length, body condition (using preinfection length and weight to calculate the scaled mass index, following Peig & Green, 2009), number of offspring, river course, river of origin (as this has only three levels, it caused model fit issues as a random effect), and how many worms initially transmitted to the fish (“dose”). To control for the effects of fish dying before day 11, we included a binary variable—“dead11”—coding whether or not the fish died before day 11, and a variable—“alivedays”—coding how many days the fish lived after either the start of the infection (for those that died before day 11), or day 11 (for those that died after day 11). Dying before day 11 cut short data collection, so we expected a positive correlation between alivedays and infection severity for these fish. However, for those that died after day 11, infection severity up to day 11 could intuitively be negatively associated with number of days they lived. We therefore included the interaction between dead11 and alivedays. As random effects, we initially included the identity of the donor used to infect the fish, and its population of origin (this term was dropped due to model fitting issues and as it explained ∼0 variance). During model simplification, number of offspring, dose, and body condition were dropped.

We addressed our second question, “do the courses differ in fecundity tolerance?,” using a generalized linear mixed model (GLMM) with a Poisson error distribution and log link function. We used the number of offspring a female was carrying as the response variable, and included as fixed effects in the full model body length, body condition, river course of origin, river of origin, how long the fish lived during infection (“infdeathdays”), infection severity, and—to test for differences in tolerance between the courses—the interaction between infection severity and river course. Body length was dropped during model simplification. The random effects were as described above in the full model, but because neither explained any variance and they caused model fit singularity, they were dropped in the final model.

We addressed our third question, “do the courses differ in mortality tolerance?,” using a GLMM with a binomial error distribution and logit link function. We used whether or not the fish survived until the end of the 17-day infection period as the response, and included as fixed effects number of offspring, body length, body condition, course, river, infection severity, and again the interaction between infection severity and river course. The number of offspring and body condition were dropped during model simplification. As random effects, we initially included population of origin and donor identity, but removed both as again they explained 0 variance and caused model fitting issues. We also addressed this question using a mixed-effects cox model looking at survival over the course of the 17-day infection, with the same fixed and random predictors as the GLMM.

## Results

**Figure 1:**
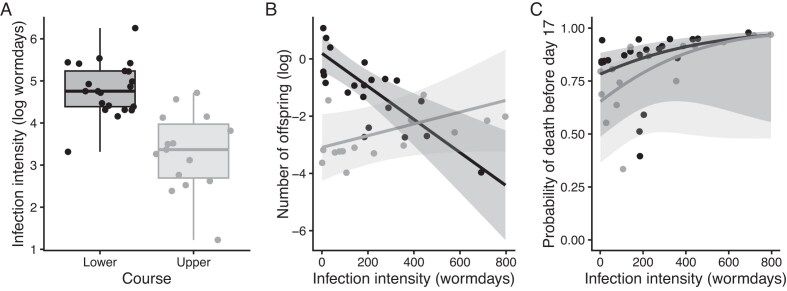
Guppy populations differ in parasite resistance (A) and fecundity tolerance (B), but not mortality tolerance (C). The points on all panels are the partial residuals from models described in the text. Panel A gives the median (thick line), interquartile range (box), and values within 1.5× the interquartile range (whiskers). Panels B and C give the model fit (lines) and standard errors (bands). There was no significant pattern in the data presented on Panel C.

Our “resistance” model revealed that fish descended from upper and lower course populations differed in the average severity of their infections (our proxy for resistance; Figure 1A; χ^2^ = 6.30, *df* = 1, *p* = 0.012). As is common in this system, larger fish had more severe infections (χ^2^ = 12.58, *df* = 1, *p* = 0.0004). Among fish that died before day 11, those that lived longer experienced higher infection severity (because of the nature of the data collection), whereas among those that died after day 11, more severe infections were associated with shorter survival times (dead11: alivedays interaction; χ^2^ = 12.13, *df* = 1, *p* = 0.0005). Fish from the Yarra River experienced more severe infections than those from the Guanapo or Aripo (χ^2^ = 6.31, *df* = 1, *p* = 0.043). Donor identity explained ∼47%, of the variance in infection severity. Partial residual plots showing the effect sizes and error associated with significant predictors in this model are in Section 4 of the supplementary material.

Our “fecundity tolerance” model showed that fish descended from upper and lower course populations differed in the relationship between infection severity and number of offspring, consistent with upper course fish being more tolerant of infection (Figure 1B; infection severity: course interaction: χ^2^ = 4.22, *df* = 1, *p* = 0.040). Post hoc tests confirm that the slope is significantly negative among lower course fish (*z* = −2.101, *p* = 0.035), and not different from 0 among upper course fish (*z* = 0.803, *p* = 0.422). Fish that lived longer (χ^2^ = 3.90, *df* = 1, *p* = 0.048) and those in better body condition (χ^2^ = 12.16, *df* = 1, *p* = 0.0005) also tended to be carrying more offspring. Partial residual plots showing the effect sizes and error associated with significant predictors in this model are in Section 5 of the supplementary material.

Our “mortality tolerance” GLMM and survival analyses both showed that there was no difference between fish descended from upper and lower course populations in how infection severity affected their probability of dying before the end of the experiment (Figure 1C; infection severity:course interaction: GLMM χ^2^ = 0.013, *df* = 1, *p* = 0.909; Cox χ^2^ = 0.820, *df* = 1, *p* = 0.365). Both models found that smaller fish were more likely to die (GLMM χ^2^ = 8.185, *df* = 1, *p* = 0.004; Cox χ^2^ = 5.40, *df* = 1, *p* = 0.020), and no other predictors explained significant portions of the variation. Full model outputs, partial residual plots of the nonfocal significant predictors in each model, and model validation plots are provided in the supplementary material.

## Discussion

Consistent with theoretical work and empirical work from other systems, our results support the maintenance of variation across populations in both parasite resistance and fecundity tolerance, but not mortality tolerance. We additionally show that investment in resistance and fecundity tolerance follow the pattern predicted by variation in predation pressure and pace of life theory across natural populations. We necessarily excluded males in this study which, because the sexes interact with these parasites differently across both ecological (Stephenson et al., 2015a, 2015b; Weiler et al., 2025) and evolutionary (Dargent et al., 2016; Kramp et al., 2024) processes, may limit our ability to extrapolate from these findings. However, that the results from this laboratory experiment concur with data from field surveys suggests that the patterns we observed here are relevant to the system in nature.

We found that upper course guppies are more resistant than lower course guppies, which is in line with some previous observations in this system (Dargent et al., 2013; Stephenson et al., 2015b) but not others (Cable & van Oosterhout, 2007; Van Oosterhout et al., 2003; using Aripo guppies, as we did here). Consistent with this variation, we found that river of origin and the identity of the donor explained substantial portions of the variance, emphasizing that maximizing sample size across these levels was important to uncover the general relationship we show here. Aspects of the donor’s identity have affected parasite growth on experimental fish in a previous study (Stephenson et al., 2017; in this case, whether the donor had transmitted before), but the mechanisms remain unclear. Overall, this result is consistent with investment in resistance that follows population differences in predator-driven life-history evolution (Ricklefs & Wikelski, 2002; Stephenson et al., 2015a; van Boven & Weissing, 2004).

Our data also test the alternative hypothesis that predator-induced increases in exposure to parasites (due to increased shoaling as an antipredator defense, and thus higher parasite transmission; Walsman et al., 2022) could select for higher investment in resistance. Despite the fact that lower course *Gyrodactylus* spp. appear to be more virulent, which should increase selection for resistance, we found that lower course guppies actually invest less in resistance than upper course guppies, possibly due to a “resistance is futile” effect (Walsman et al., 2023). Further, that we found variation between populations in resistance suggests that it is costly: costs of resistance are a key ingredient which, combined with negative frequency dependent selection, permits the maintenance of variation such as that we observe (Antonovics & Thrall, 1994; Best et al., 2014; Boots & Bowers, 2004). This result supports previous experimental work: when mated to parasite naive females, more resistant male guppies produced offspring slower than their less resistant counterparts (Weiler et al., 2025). Such delayed reproduction would be especially detrimental to lower course guppies that experience higher extrinsic mortality from both predators and parasites (Walsman et al., 2022). Overall, the fact that lower course guppies invest less in resistance and fecundity tolerance than upper course guppies, despite their local *Gyrodactylus* spp. being more virulent and prevalent, suggests that the cost of infection is likely substantial in these populations.

While the patterns we observe are in line with evolutionary predictions, the extent to which the variation we found is genetic is unclear: all three defense metrics likely have some degree of plasticity. In other systems, fecundity responds to resource availability (Pollux & Reznick, 2011) and to infection in nuanced ways: infection can result in lower (Jehan et al., 2021; Weiler et al., 2025), higher (Schwanz, 2008), or ecomorph-dependent changes (Firkus et al., 2022) in reproductive output. Similarly, host immune investment responds plastically to exposure to predators (Navarro et al., 2004). However, while we observe similar plasticity in this system (Reznick & Yang, 1993; Weiler et al., 2025), we know that both resistance (Dargent et al., 2013; Kramp et al., 2024; Madhavi & Anderson, 1985; Weiler et al., 2025) and tolerance (Kramp et al., 2024; quantified using body condition as a proxy for fitness) are heritable, and we kept experimental conditions as consistent as possible.

We present our fecundity tolerance result as infection affecting fecundity, but we cannot rule out the effects of pregnancy on immune function. Indeed, pregnancy represents a life-history state in which the costs of immune activation increase (Graham et al., 2011; Råberg et al., 2009). A potential alternative explanation of our fecundity tolerance result is therefore that females vary in their ability to acquire resources: some lower course guppies appear able to invest in both reproduction and immunity, keeping parasite loads low while producing many offspring (van Noordwijk & de Jong, 1986). We did find that females carrying more offspring lived longer, potentially further corroborating the idea that some females were overall higher quality, and able to carry larger broods while both resisting and tolerating infection. That we found a positive correlation with body condition is also supportive of this idea, but as guppies are livebearers (so female mass encompasses both somatic body condition and offspring mass), we are cautious in interpreting this result. It is also not consistent with this explanation that the population-level differences we observed persisted under common garden conditions.

Guppy biology is such that we could not know what stage of their reproductive cycle females were in when we infected them. Female guppies are lecithotrophic: embryos are nourished by yolk before birth, with no additional maternal provisioning during development (Houde, 1997). Instead, female guppies invest shortly before parturition: a new batch of ova matures and is fertilized after a brood is born (Houde, 1997). This investment is substantial: reproducing females allocate approximately twice as much energy to reproductive tissues at the expense of somatic growth compared to virgins (Reznick, 1983). In our experiment, females were taken from mixed-sex breeding tanks, and all were sexually mature adults. It is highly likely that they had all mated with at least one male before being isolated and infected. However, as females store sperm (Magurran, 2005), and exhibit cryptic female choice (Gasparini & Pilastro, 2011), they likely control when and how many eggs are fertilized, and potentially embryo developmental speed (Sato et al., 2021). It is therefore almost impossible to know at which stage in their reproductive cycle they were when infected, and likely would be even had we employed a controlled mating design.

Dissecting the females increased the accuracy of our fecundity metric, but it remains imperfect. Females under stress can prematurely birth offspring (Ord et al., 2020), and newborn fish are vulnerable to cannibalism, particularly in small tanks such as those we used (Riesch et al., 2022). Despite females being isolated and monitored daily for at least 20 days, we only noted one birth during the experiment (removing this female did not change our results), potentially indicating that additional births were missed due to cannibalism. Further, while there is no evidence for selective abortion in guppies or even matrotrophic poecilids under starvation (Banet & Reznick, 2008; Meffe & Vrijenhoek, 1981), abortion and resorption under the stress of infection in this experiment may have allowed females to divert resources toward immunity.

Overall, our results support the role of epidemiological feedback in the evolution of host defenses, and highlight that host defense evolution is additionally subject to selection from interactions with the broader ecological community.

## Supplementary Material

qrag011_Supplemental_File

## Data Availability

The data are archived on Dryad at https://doi.org/10.5061/dryad.0zpc867bf.
