# Supplementary material for: Pace of life predicts parasite resistance and fecundity tolerance, but not mortality tolerance, among Trinidadian guppies, Poecilia reticulata
Source: Evol Lett. 2026 Mar 31;10(3):294–300. doi: 10.1093/evlett/qrag011 (PMC13229650; doi:10.1093/evlett/qrag011)
Supplement: qrag011_Supplemental_File [file qrag011_supplemental_file.pdf]

Pace of life predicts Trinidadian guppy parasite resistance and fecundity tolerance, but not mortality tolerance

S. Evanov, F. Rovenolt, N. Tepox Vivar, R. Zambetti, K. Troutman, V. Nayak, K. Mulligan, S. Midde, N. T. Leith, J. F. Stephenson

Contents

1 Introduction **1**

2 Import data and create new data frames **2**

3 Checking the data **2**

4 Question 1: Do the courses differ in resistance? **3**
4.1 Justification for truncating infection severity at day 11 3
4.2 Analysis 4

5 Question 2: Do the courses differ in fecundity tolerance? **9**

6 Question 3: Do the courses differ in mortality tolerance? **13**
6.1 GLMM 13
6.2 Survival analysis 16

1 Introduction

We used the datasheet 'Inf_Dissections_Trini.csv' in this analysis, which contains the following variables:

MaternalID: Identify of the mother
DateDissected: Date sample was dissected in YYYYMMDD format **OrderDissected:** For each day of dissection, order dissection occurred in (1 = 1st sample dissected on that date, 2 = 2nd, etc.)
OffspringNumber: Number of offspring; since each offspring gets its own row, should be either 1 (with a stage in the next column) or 0 (mother had no offspring)
OffspringDevelopmentalStage: Development stage (A-J) of offspring
offstage: As above, but with letters converted to numbers - A=1, B=2.. etc. If no offspring were found, NA.
offstage0: As above, but with letters converted to numbers - A=1, B=2.. etc. If no offspring were found, 0.
DissectorInitials: Initials of dissectors (CW = Charlie Walsh, NT = Natalie Tepox)
Batch: Infections were conducted in batches 1-3
Population: Population of fish
River: River the progenitors of these fish were originally collected from **Course:** River course of collection **AUC:** Area under the curve of parasite over time
D11AUC: Area under the curve of parasite over time up to day 11 of infection
InfDeathDays: Number of days between infection and death (including euthanasia at end of experiment)
dead11: Whether or not the fish was dead before day 11 counts; 'yes' they were, or 'no', they lived at least until the day 11 counts.
alivedays: The number of days the fish was alive before day 11 (if it died before day 11), or after day 11 (if it died after day 11). This variable - along with dead11 - helps us to account for potentially different patterns

of how D11AUC was related to the length of time the fish lived.
PreDate: Date in YYYYMMDD of weighing and lengthing fish before infection
PreWeight: Weight of fish in grams before infection
PreLength: Length of fish in centimeters before infection
PreSMI: Scaled mass length from before infection based on linear model predicting weight by length using preinfection metrics
PostDate: Date in YYYYMMDD of weighing and lengthing fish fater infection
PostWeight: Weight of fish in grams after infection
PostLength: Length of fish in centimeteres after infection
PostSMI: Scaled mass index from after infection based on linear model predicting weight by length using preinfection metrics
DeltaSMI: PostSMI - PreSMI
DoseDate: Day fish was infected in YYYYMMDD
Dose: Total number of worms on fish for initial infection
DeathDate: Day of death in YYYYMMDD–for fish that lasted until the last day of the experiment, date of the last day and when they were euthanized
PrematureDeath: Y if fish died before end of eexperiment; N if fish was euathanized at end of experiment

2 Import data and create new data frames

Our datasheet is organized so that each offspring has its own row. Here, we use the raw data to add a variable:

totaloffspring: adds all offspring attributed to that unique maternal ID to give a straightforward count of offspring.

We then create a new dataframe with only one row per mother.

```
DFIMP <- read.csv("Inf_Dissections_Trini.csv")
DFIMP$Maternal_ID <- as.factor(DFIMP$Maternal_ID)

ocount <- ddply(DFIMP, c("Maternal_ID"),summarise,
                   totaloffspring = sum(Offspring_Number))

dfall<-merge(ocount, DFIMP, by="Maternal_ID")

#data with one observation
dfu <- dfall %>%
  arrange(Maternal_ID) %>%
distinct(Maternal_ID, .keep_all = TRUE)

#standardise
```

3 Checking the data

Looking at the correlations among variables to confirm our predictors are ok to include in the same model.

```
ggpairs(dfu,
             columns = c("AUC", "D11AUC", "InfDeathDays",
                         "totaloffspring",
                         "Course", "PreSMI", "PreLength","dead11"))
```

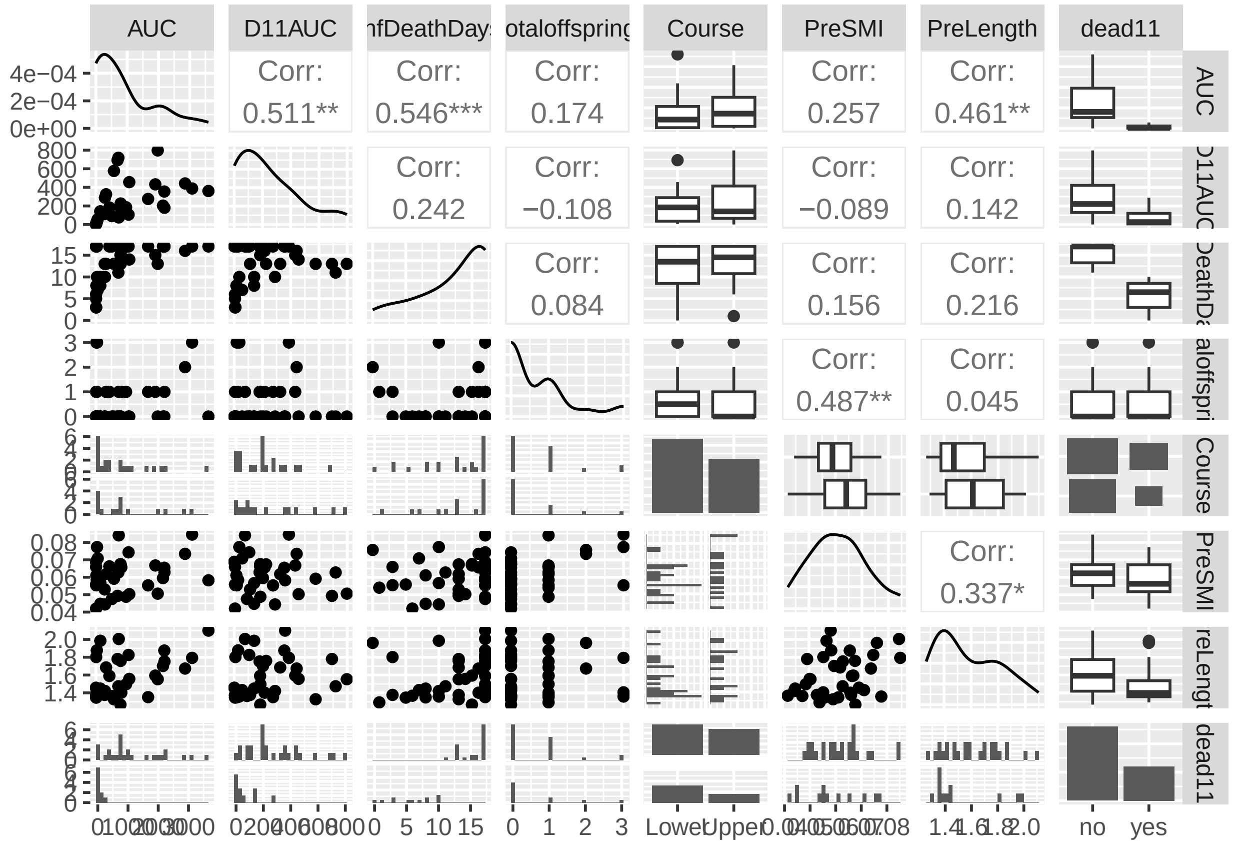

4 Question 1: Do the courses differ in resistance?

4.1 Justification for truncating infection severity at day 11

We truncated our infection severity variable at day 11 because this day represents a good balance between ensuring sufficient parasite counts for a robust metric, and minimising the number of fish with missing data due to premature death. Here is a histogram of the InfDeathDays of these fish, with an overlay of the cumulative data. The red line denotes day 11.

```
## Warning: The dot-dot notation (`..count..`) was deprecated in ggplot2 3.4.0.
## i Please use `after_stat(count)` instead.
## This warning is displayed once every 8 hours.
## Call `lifecycle::last_lifecycle_warnings()` to see where this warning was
## generated.
```

```
## `stat_bin()` using `bins = 30`. Pick better value `binwidth`.
```

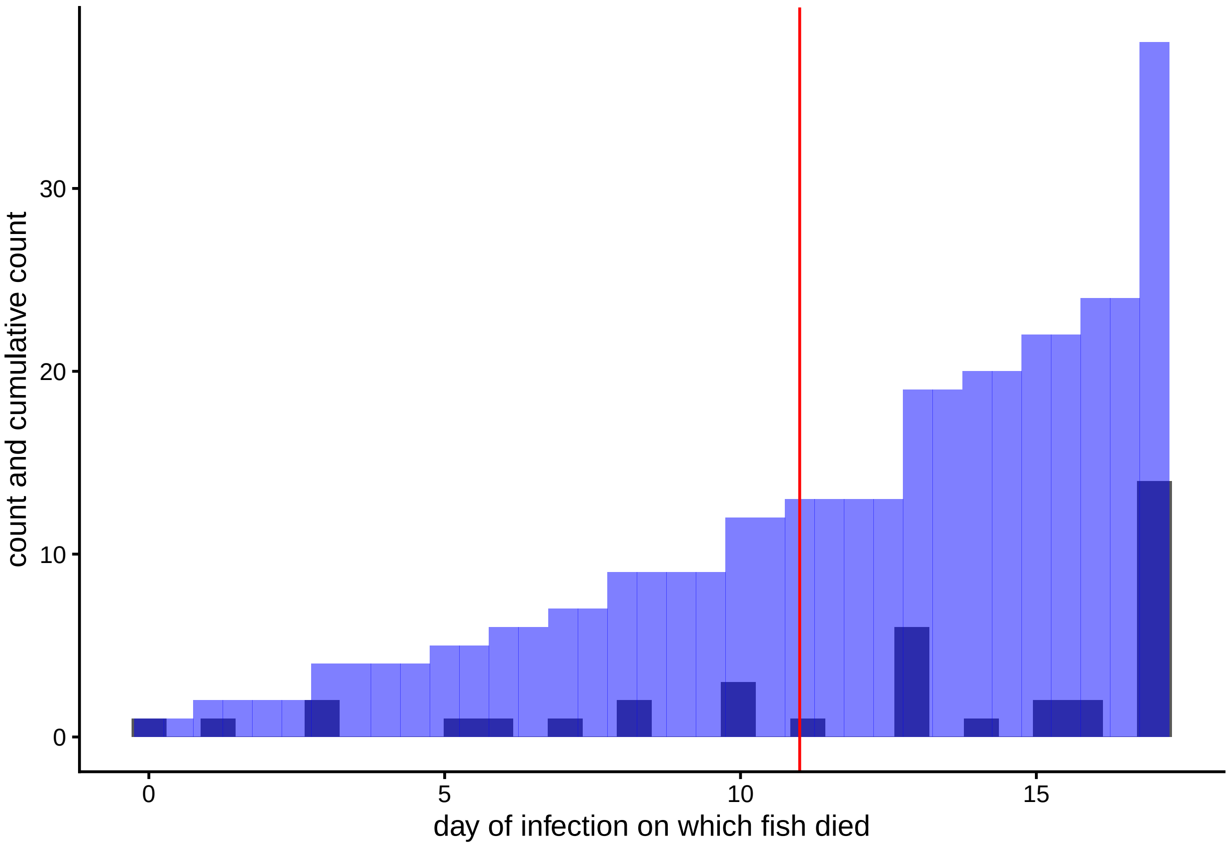

4.2 Analysis

Lower courses invest less in resistance/get higher parasite loads.

In this analysis, we included all variables that we a priori thought may affect the infection integral of these fish. We used drop1 to optimise this list, and report the final model in the main text. Here, we leave the original terms in the model, commented out, to show what we originally included. We include River as a fixed effect because we only have data from 3 rivers; our interest here is mostly how much variation there might be between rivers, so ideally we would include this as a random term. However, doing so worsens the model fit (singularity issues).

```
#dfu2<-subset(dfu, "Maternal_ID"!="B1_UY_F4")# to remove the female
#that gave birth to 1 offspring during her infection

M1 <- lmer(log(D11AUC) ~ PreLength+
                    #totaloffspring+
                    Course+
                    River+
                    #Dose+
                    #PreSMI+
                    alivedays*
                    dead11+
                    (1|DonorID),
                    #(1|Population),
           data = dfu, na.action=na.omit)

summary(M1)
```

```
## Linear mixed model fit by REML ['lmerMod']
## Formula: log(D11AUC) ~ PreLength + Course + River + alivedays * dead11 +
##     (1 | DonorID)
##    Data: dfu
##
## REML criterion at convergence: 101
##
## Scaled residuals:
##      Min       1Q   Median       3Q      Max
## -2.30081 -0.55797 -0.00093  0.58127  1.58165
##
## Random effects:
##  Groups   Name        Variance Std.Dev.
##  DonorID  (Intercept) 0.7383   0.8592
##  Residual             0.8228   0.9071
## Number of obs: 36, groups:  DonorID, 6
##
## Fixed effects:
##                      Estimate Std. Error t value
## (Intercept)          1.689500   1.396157   1.210
## PreLength            3.134357   0.883701   3.547
## CourseUpper         -1.508225   0.600796  -2.510
## RiverGuanapo        -0.007931   0.487303  -0.016
## RiverYarra           1.417001   0.565324   2.507
## alivedays           -0.275042   0.109433  -2.513
## dead11yes           -5.260375   1.095070  -4.804
## alivedays:dead11yes  0.620445   0.178127   3.483
##
## Correlation of Fixed Effects:
##             (Intr) PrLngt CrsUpp RvrGnp RvrYrr alvdys dd11ys
## PreLength   -0.877
## CourseUpper  0.138 -0.256
## RiverGuanap  0.144 -0.338  0.410
## RiverYarra  -0.064  0.011 -0.655  0.065
## alivedays   -0.193 -0.100 -0.247 -0.173  0.220
## dead11yes   -0.240  0.060 -0.148 -0.111  0.195  0.531
## alvdys:dd11  0.191 -0.014  0.265  0.265 -0.218 -0.723 -0.915
```

```r
Anova(M1)
```

```
## Analysis of Deviance Table (Type II Wald chisquare tests)
##
## Response: log(D11AUC)
##                    Chisq Df Pr(>Chisq)
## PreLength        12.5802  1  0.0003899 ***
## Course            6.3020  1  0.0120602 *
## River             6.3148  2  0.0425371 *
## alivedays         0.0000  1  0.9950114
## dead11           16.0634  1  6.126e-05 ***
## alivedays:dead11 12.1324  1  0.0004955 ***
## ---
## Signif. codes:  0 '***' 0.001 '**' 0.01 '*' 0.05 '.' 0.1 ' ' 1
```

```r
drop1(M1, test="Chisq")
```

```
## Single term deletions
##
## Model:
## log(D11AUC) ~ PreLength + Course + River + alivedays * dead11 +
##     (1 | DonorID)
##                 npar    AIC     LRT   Pr(Chi)
## <none>               116.80
## PreLength          1 124.78  9.9742 0.0015875 **
## Course             1 122.10  7.2960 0.0069109 **
## River              2 119.86  7.0551 0.0293762 *
## alivedays:dead11   1 127.66 12.8631 0.0003351 ***
## ---
## Signif. codes:  0 '***' 0.001 '**' 0.01 '*' 0.05 '.' 0.1 ' ' 1
```

```r
simulateResiduals(M1, plot = T)
```

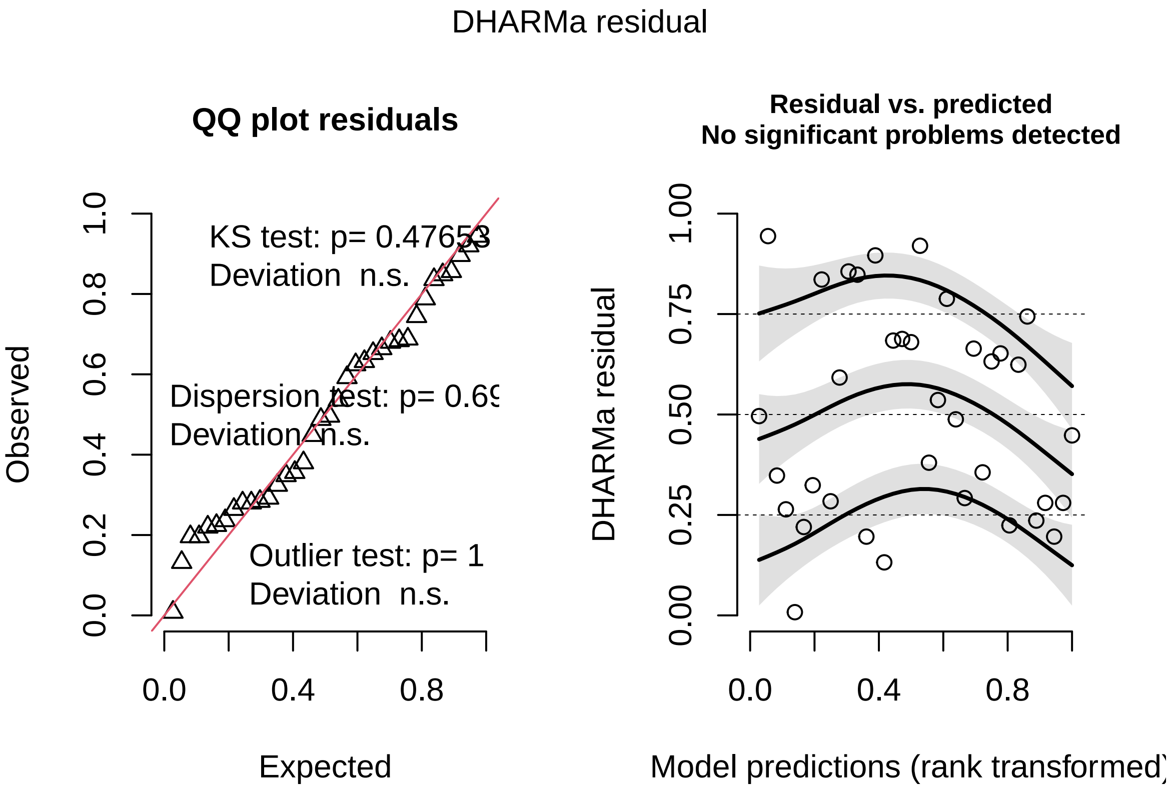


```
## Object of Class DHARMa with simulated residuals based on 250 simulations with refit = FALSE . See ?DH
##
## Scaled residual values: 0.292 0.836 0.324 0.536 0.448 0.68 0.28 0.488 0.224 0.664 0.264 0.196 0.624 0
```

```r
vif(M1)
```

```
##                    GVIF Df GVIF^(1/(2*Df))
## PreLength      1.291828  1        1.136586
## Course         2.921856  1        1.709344
## River          3.810154  2        1.397126
## alivedays      2.835095  1        1.683774
## dead11        10.128124  1        3.182471
```

```
## alivedays:dead11 15.534327  1        3.941361
```

```r
#variance calculation - from the above model
m1donor<- 0.7383
m1resid<- 0.8228
m1pop<- 0.0000 # this is the value when Population is included as random term in the above model

m1tot<-m1donor+m1resid

m1donor/m1tot
```

```
## [1] 0.4729358
```

```r
m1pop/m1tot
```

```
## [1] 0
```

```r
m1resid/m1tot
```

```
## [1] 0.5270642
```

```r
visreg(M1, "River", scale="response", partial=T)
```

```
## Warning:   Note that you are attempting to plot a 'main effect' in a model that contains an
##   interaction.  This is potentially misleading; you may wish to consider using the 'by'
##   argument.

## Conditions used in construction of plot
## PreLength: 1.525
## Course: Lower
## alivedays: 6
## dead11: no
## DonorID: CAGT1_Pop5_D3
```

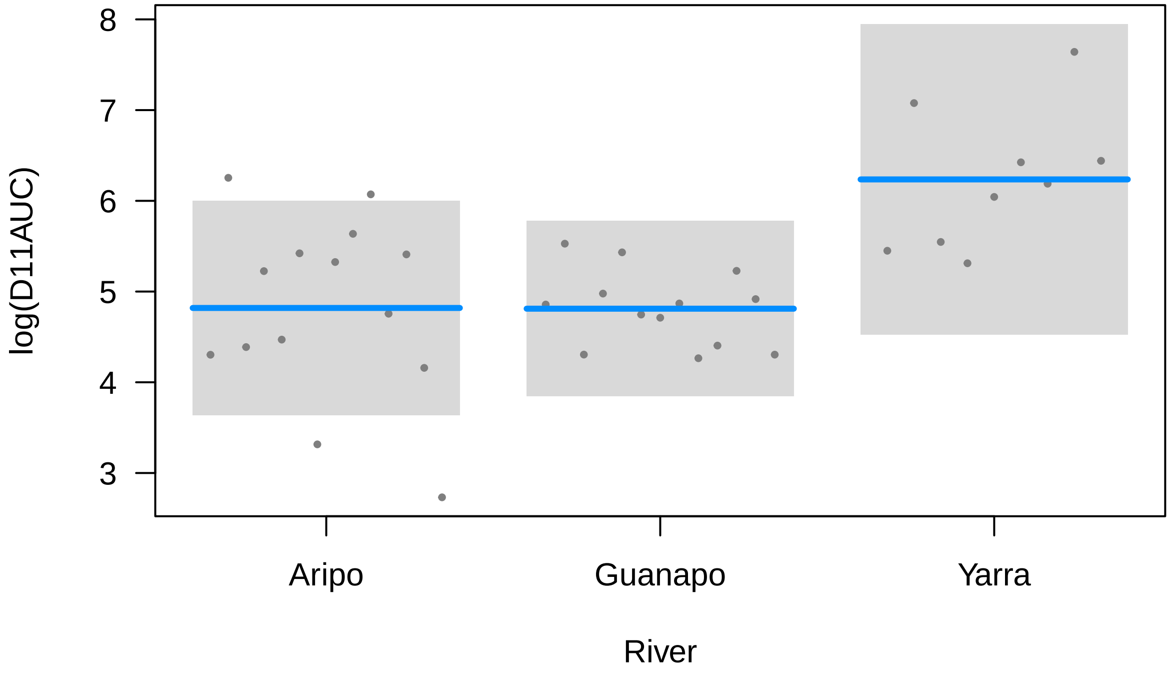


```r
visreg(M1, "PreLength", scale="response", partial=T)
```

```
## Warning:   Note that you are attempting to plot a 'main effect' in a model that contains an
```

```
##   interaction.  This is potentially misleading; you may wish to consider using the 'by'
##   argument.
```

```
## Conditions used in construction of plot
## Course: Lower
## River: Aripo
## alivedays: 6
## dead11: no
## DonorID: CAGT1_Pop5_D3
```

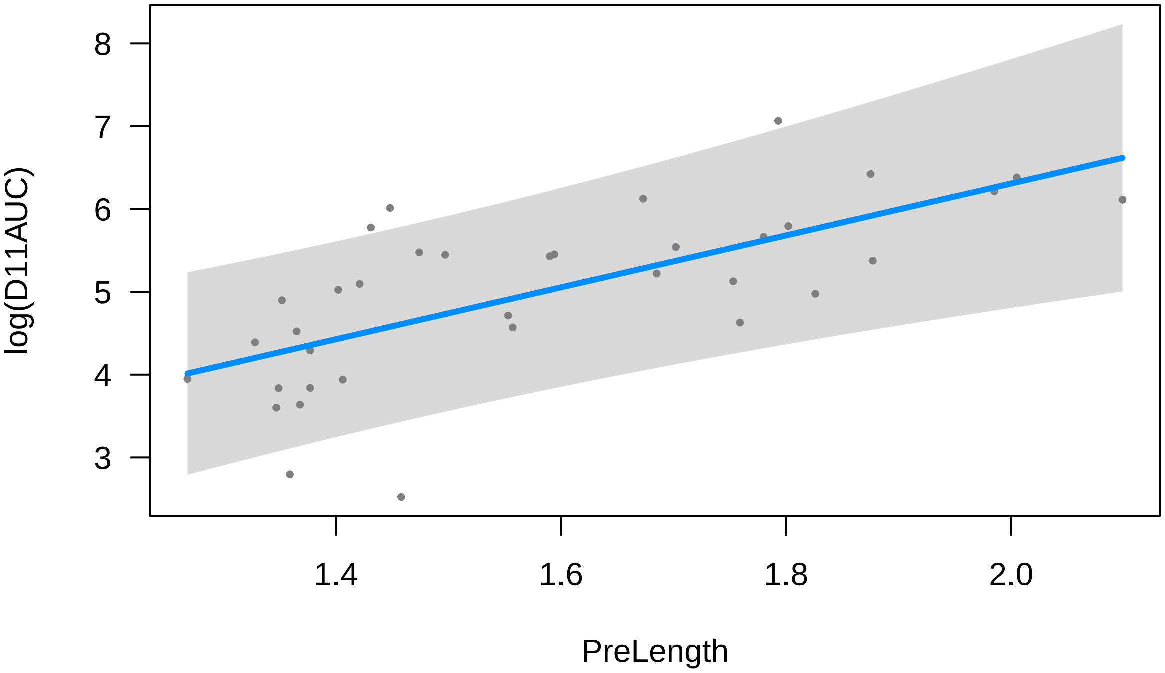


```r
visreg(M1, "alivedays", by="dead11", scale="response", partial=T)
```

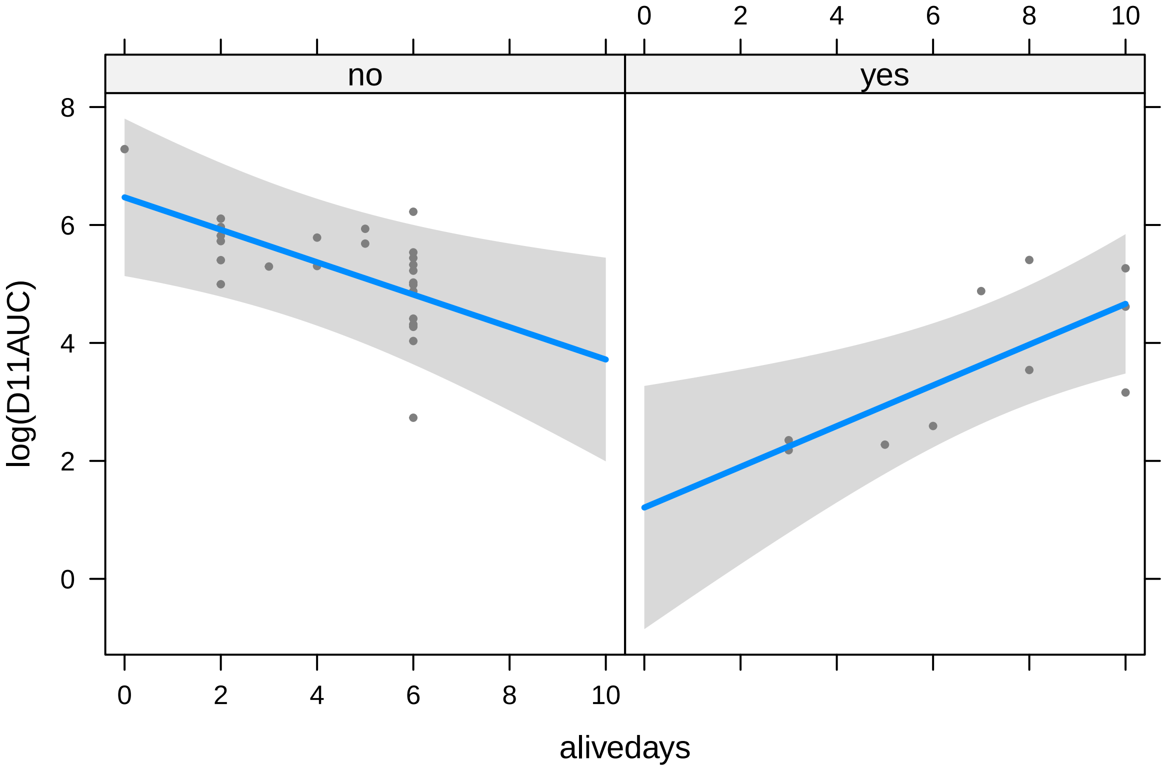

5 Question 2: Do the courses differ in fecundity tolerance?

For this analysis - it's at the level of the individual mother - we're testing essentially "are there maternal attributes such as her population of origin, her infection severity, her size.. etc that predict the number of offspring we found in her?"

For this analysis, as above, we chose predictors that we a priori thought should be important in predicting a female's total offspring. We used drop1 to optimise this list, and report the final model in the main text. We leave all variables in this code, commented out, to show what was originally included and removed during the model simplification process. While we originally included random effects to account for data structure, we removed these as they explained 0 variance and dramatically worsened model fits. Drop1 suggests removing River would reduce AIC slightly, but we retain this term to account for the fact that these fish came from different rivers.

```
M2 <- glm(totaloffspring ~ #PreLength +
                    PreSMI+
                     Course+
                    D11AUC+
                    Course:D11AUC+
                    InfDeathDays+
                    River, #retained to account for data structure
              #(1|DonorID)+
              #(1|Population),
                   family=poisson(link="log"), data = dfu, na.action=na.omit)
summary(M2)
```

```
##
## Call:
## glm(formula = totaloffspring ~ PreSMI + Course + D11AUC + Course:D11AUC +
##     InfDeathDays + River, family = poisson(link = "log"), data = dfu,
```

```
##     na.action = na.omit)
##
## Coefficients:
##                      Estimate Std. Error z value Pr(>|z|)
## (Intercept)         -7.493107   2.172397  -3.449 0.000562 ***
## PreSMI              90.896512  28.156432   3.228 0.001245 **
## CourseUpper         -3.287347   1.106665  -2.970 0.002973 **
## D11AUC              -0.005776   0.002750  -2.101 0.035679 *
## InfDeathDays         0.152237   0.083928   1.814 0.069694 .
## RiverGuanapo         0.681214   0.646637   1.053 0.292125
## RiverYarra           0.719042   1.081503   0.665 0.506143
## CourseUpper:D11AUC  0.007833   0.003891   2.013 0.044122 *
## ---
## Signif. codes:  0 '***' 0.001 '**' 0.01 '*' 0.05 '.' 0.1 ' ' 1
##
## (Dispersion parameter for poisson family taken to be 1)
##
##     Null deviance: 45.185  on 35  degrees of freedom
## Residual deviance: 20.293  on 28  degrees of freedom
##   (2 observations deleted due to missingness)
## AIC: 67.882
##
## Number of Fisher Scoring iterations: 5
```

```
Anova(M2)
```

```
## Analysis of Deviance Table (Type II tests)
##
## Response: totaloffspring
##                LR Chisq Df Pr(>Chisq)
## PreSMI          12.1552  1  0.0004895 ***
## Course           7.6314  1  0.0057360 **
## D11AUC           1.3072  1  0.2529019
## InfDeathDays     3.9029  1  0.0482016 *
## River            1.3294  2  0.5144202
## Course:D11AUC   4.2225  1  0.0398916 *
## ---
## Signif. codes:  0 '***' 0.001 '**' 0.01 '*' 0.05 '.' 0.1 ' ' 1
```

```
drop1(M2, test="Chisq")
```

```
## Single term deletions
##
## Model:
## totaloffspring ~ PreSMI + Course + D11AUC + Course:D11AUC + InfDeathDays +
##     River
##                Df Deviance    AIC     LRT  Pr(>Chi)
## <none>              20.293 67.882
## PreSMI          1   32.448 78.038 12.1552 0.0004895 ***
## InfDeathDays    1   24.196 69.785  3.9029 0.0482016 *
## River           2   21.623 65.212  1.3294 0.5144202
## Course:D11AUC   1   24.516 70.105  4.2225 0.0398916 *
## ---
## Signif. codes:  0 '***' 0.001 '**' 0.01 '*' 0.05 '.' 0.1 ' ' 1
```

```r
simulateResiduals(M2, plot = T)
```

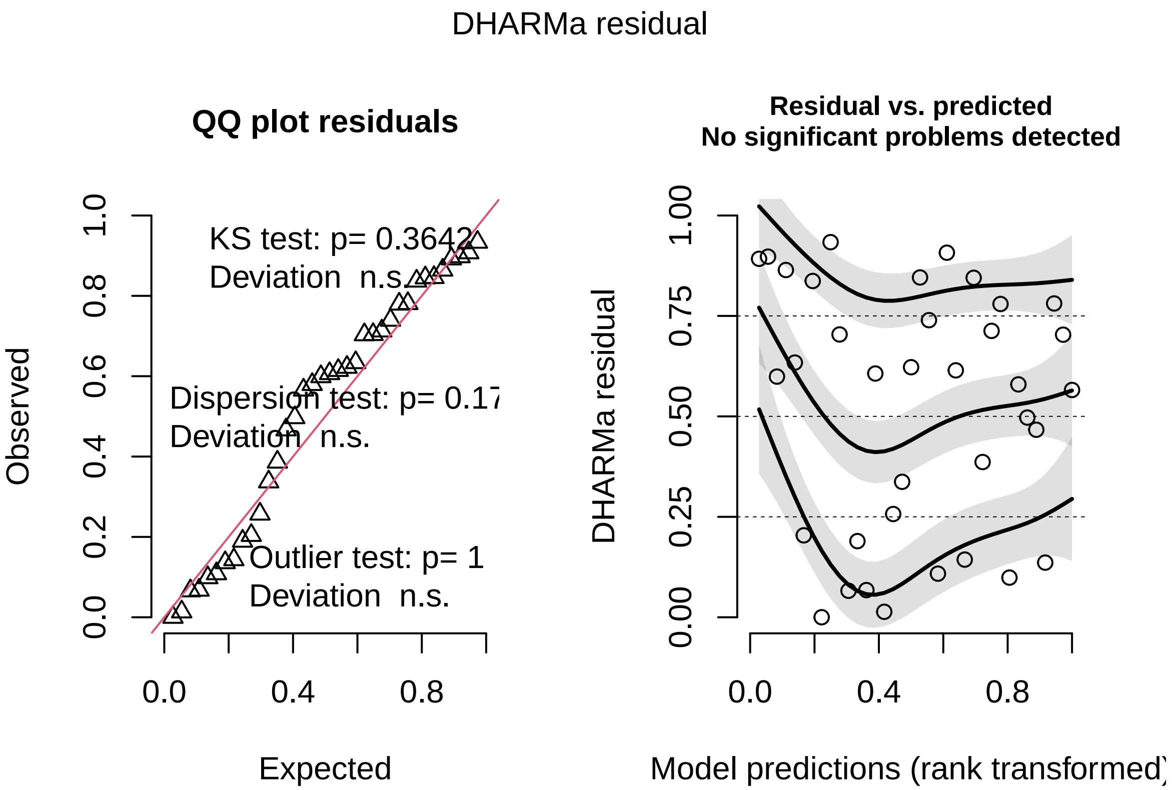


```
## Object of Class DHARMa with simulated residuals based on 250 simulations with refit = FALSE . See ?DI
##
## Scaled residual values: 0.622412 0.8369375 0.1435377 0.5796685 0.8975354 0.614558 0.1085635 0.098734
```

```r
visreg(M2, "InfDeathDays", scale="response", partial=T)
```

```
## Conditions used in construction of plot
## PreSMI: 0.06030244
## Course: Lower
## D11AUC: 184
## River: Aripo
```

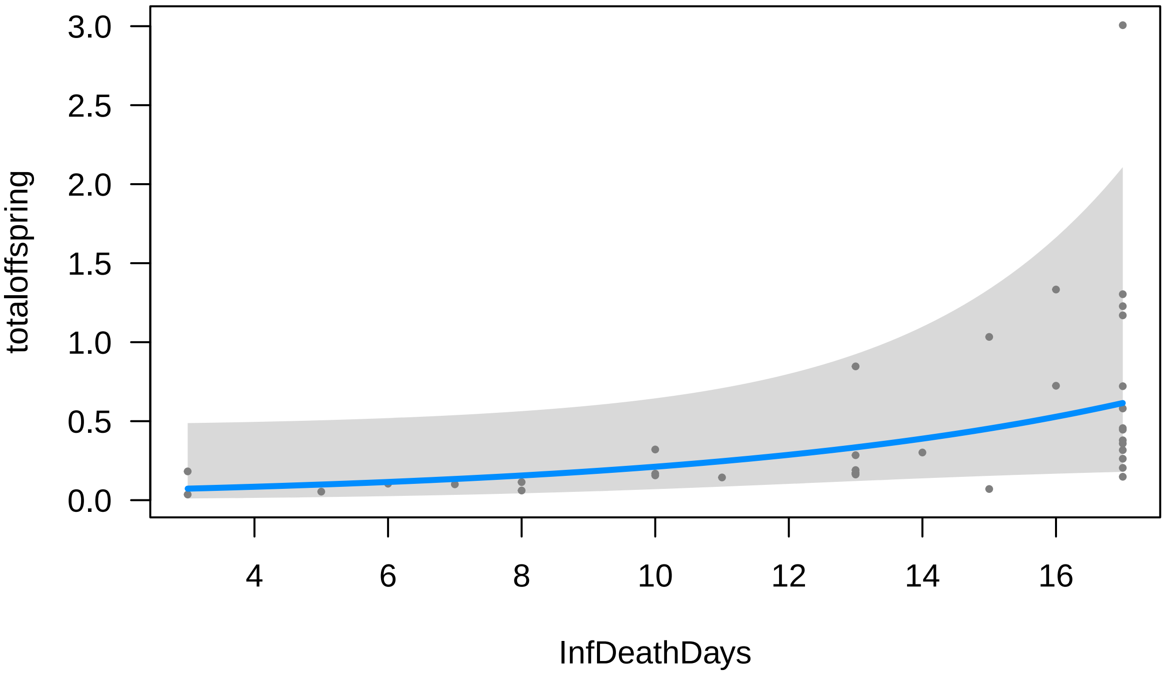


```r
visreg(M2, "PreSMI", scale="response", partial=T)
```

```
## Conditions used in construction of plot
## Course: Lower
## D11AUC: 184
## InfDeathDays: 14.5
## River: Aripo
```

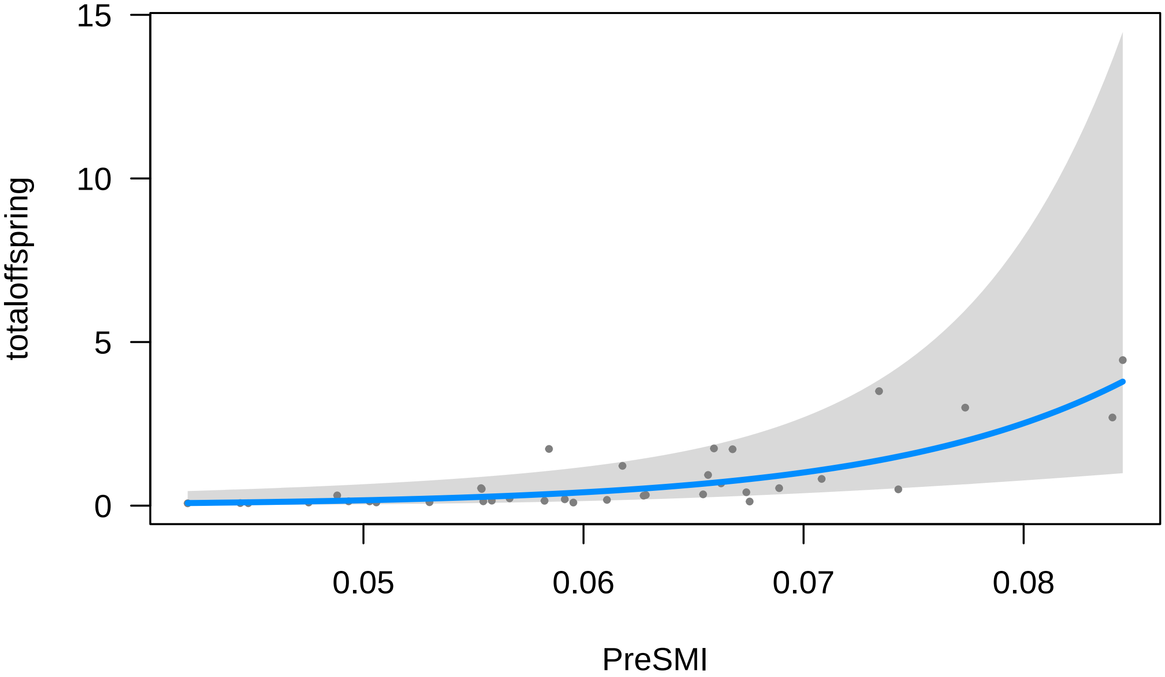


```r
#post hoc test
M2.post <- test(emtrends(M2, specs = "Course", var = "D11AUC"))
summary(M2.post)
```

```
##  Course D11AUC.trend      SE  df z.ratio p.value
##  Lower      -0.00578 0.00275 Inf  -2.101  0.0357
##  Upper       0.00206 0.00256 Inf   0.803  0.4221
##
## Results are averaged over the levels of: River
```

6 Question 3: Do the courses differ in mortality tolerance?

6.1 GLMM

Overall no - there is no difference in probability of dying, and no difference in how infection loads contribute to this probability.

Again, we started off including all variables about which we had a priori ideas for how they could influence the probability of dying. We used drop1 to optimise this list, but stopped removing terms once those that we wanted to retain because they were focal to this analysis or due to data structure remained. We also initially included random terms, but these explained almost none of the variance and made for singular model fits, so we removed them.

```r
#Creating new variable of whether or not they died prematurely
dfu$PD <- ifelse(dfu$PrematureDeath == "Y", 1, 0)  #

M3<-glm(PD~
              #totaloffspring+
              #PreSMI+
              Course+
              D11AUC+
              PreLength+
              D11AUC:Course+ #retained as focal term
              River, #retained due to data structure
            #(1|DonorID)+
            #(1|Population),
            data=dfu,
            family=binomial(link="logit"))

summary(M3)
```

```
##
## Call:
## glm(formula = PD ~ Course + D11AUC + PreLength + D11AUC:Course +
##     River, family = binomial(link = "logit"), data = dfu)
##
## Coefficients:
##                      Estimate Std. Error z value Pr(>|z|)
## (Intercept)        14.2080634  5.6259677   2.525   0.0116 *
## CourseUpper        -0.6456615  1.9198438  -0.336   0.7366
## D11AUC              0.0026548  0.0037942   0.700   0.4841
## PreLength          -8.4793343  3.8744204  -2.189   0.0286 *
## RiverGuanapo        0.3049297  1.6826833   0.181   0.8562
## RiverYarra          2.6994683  1.9781774   1.365   0.1724
## CourseUpper:D11AUC  0.0007778  0.0068739   0.113   0.9099
## ---
## Signif. codes:  0 '***' 0.001 '**' 0.01 '*' 0.05 '.' 0.1 ' ' 1
##
## (Dispersion parameter for binomial family taken to be 1)
```

```
##
##     Null deviance: 38.139  on 35  degrees of freedom
## Residual deviance: 23.013  on 29  degrees of freedom
##   (2 observations deleted due to missingness)
## AIC: 37.013
##
## Number of Fisher Scoring iterations: 6
```

```
Anova(M3)
```

```
## Analysis of Deviance Table (Type II tests)
##
## Response: PD
##                LR Chisq Df Pr(>Chisq)
## Course           0.1041  1   0.746961
## D11AUC           1.0278  1   0.310685
## PreLength        8.1849  1   0.004224 **
## River            2.3628  2   0.306846
## Course:D11AUC    0.0131  1   0.908987
## ---
## Signif. codes:  0 '***' 0.001 '**' 0.01 '*' 0.05 '.' 0.1 ' ' 1
```

```
drop1(M3, test="Chisq")
```

```
## Single term deletions
##
## Model:
## PD ~ Course + D11AUC + PreLength + D11AUC:Course + River
##               Df Deviance    AIC    LRT Pr(>Chi)
## <none>             23.013 37.013
## PreLength      1   31.198 43.198 8.1849 0.004224 **
## River          2   25.376 35.376 2.3628 0.306846
## Course:D11AUC  1   23.026 35.026 0.0131 0.908987
## ---
## Signif. codes:  0 '***' 0.001 '**' 0.01 '*' 0.05 '.' 0.1 ' ' 1
```

```
simulateResiduals(M3, plot = T)
```

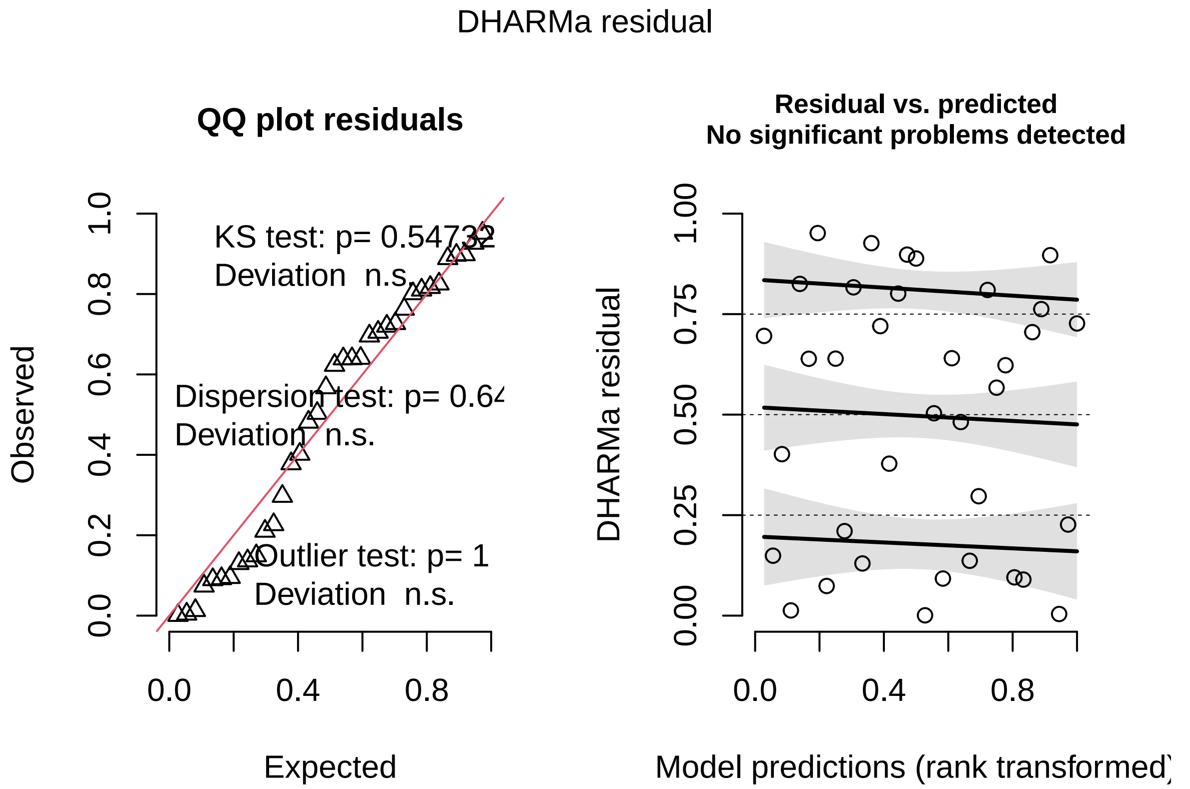


```
## Object of Class DHARMa with simulated residuals based on 250 simulations with refit = FALSE . See ?DI
##
## Scaled residual values: 0.8100863 0.8883216 0.297005 0.1298107 0.9264852 0.0007870627 0.1491113 0.073
```

```r
# more likely to die if smaller
visreg(M3, "PreLength", scale = "response", partial = T)
```

```
## Conditions used in construction of plot
## Course: Lower
## D11AUC: 184
## River: Aripo
```

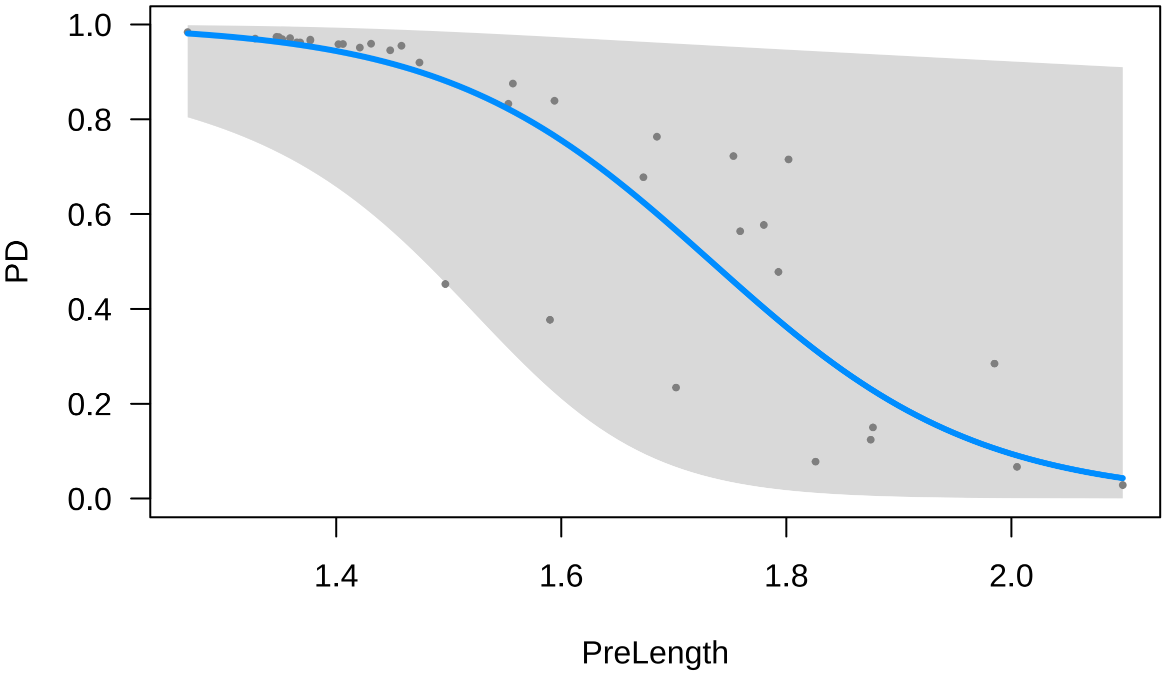

6.2 Survival analysis

```
library(survival)
library(survminer)
library(coxme)
library(ade4)

#Analysis type Kaplan/Meier o Cox Model

dfu$surv_time <- dfu$InfDeathDays
dfu$status <- ifelse(dfu$InfDeathDays < 17, 1, 0)  # 1 = murio antes del dia 17,
#0 = censurada (viva hasta el final)

cox_dfio <- coxph(Surv(surv_time, status) ~ Course*D11AUC, data = dfu)
summary(cox_dfio)
```

```
## Call:
## coxph(formula = Surv(surv_time, status) ~ Course * D11AUC, data = dfu)
##
##   n= 36, number of events= 22
##    (2 observations deleted due to missingness)
##
##                           coef exp(coef)  se(coef)      z Pr(>|z|)
## CourseUpper         -1.304855  0.271212  0.788611 -1.655    0.098 .
## D11AUC              -0.001685  0.998316  0.001991 -0.846    0.397
## CourseUpper:D11AUC  0.003585  1.003591  0.002411  1.486    0.137
## ---
## Signif. codes:  0 '***' 0.001 '**' 0.01 '*' 0.05 '.' 0.1 ' ' 1
##
##                    exp(coef) exp(-coef) lower .95 upper .95
## CourseUpper           0.2712     3.6872   0.05782     1.272
## D11AUC                0.9983     1.0017   0.99443     1.002
```

```
## CourseUpper:D11AUC    1.0036     0.9964   0.99886     1.008
##
## Concordance= 0.628  (se = 0.079 )
## Likelihood ratio test= 3.22  on 3 df,   p=0.4
## Wald test            = 2.98  on 3 df,   p=0.4
## Score (logrank) test = 3.08  on 3 df,   p=0.4
```

```
Anova(cox_dfio)
```

```
## Analysis of Deviance Table (Type II tests)
##                LR Chisq Df Pr(>Chisq)
## Course          0.63701  1     0.4248
## D11AUC          0.27140  1     0.6024
## Course:D11AUC   2.35316  1     0.1250
```

```
ggsurvplot(survfit(Surv(surv_time, status) ~ Course, data = dfu),
           data = dfu,
           risk.table = TRUE,
           pval = TRUE,
           xlab = "Post Infection Days",
           ylab = "Survival Probability",
           palette = "Dark2")
```

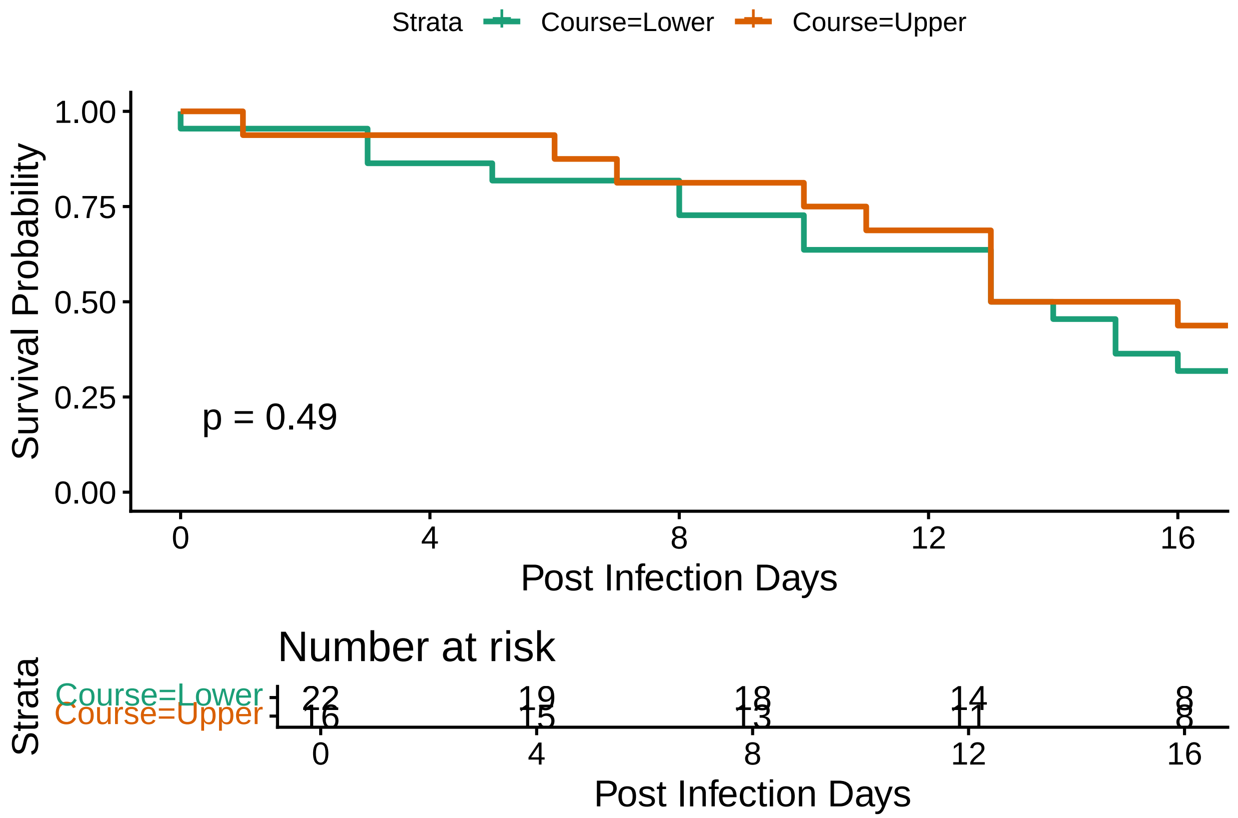


```
# Can use a mixed effects cox model with the same predictors as the binomial mortality glm
coxme_dfio <- coxme(Surv(surv_time, PD) ~ totaloffspring +
                      Course +
                      D11AUC +
                      PreLength +
                      D11AUC:Course +
```

```
                        (1|Population) + (1|River), data = dfu)
Anova(coxme_dfio) # same results
```

```
## Analysis of Deviance Table (Type II tests)
##
## Response: Surv(surv_time, PD)
##                 Df  Chisq Pr(>Chisq)
## totaloffspring  1 0.7988    0.37145
## Course          1 0.1102    0.73992
## D11AUC          1 0.6683    0.41365
## PreLength       1 5.3966    0.02018 *
## Course:D11AUC   1 0.8203    0.36510
## ---
## Signif. codes:  0 '***' 0.001 '**' 0.01 '*' 0.05 '.' 0.1 ' ' 1
```
